# Eliminating the White Supremacy Mindset from Global Health Education

**DOI:** 10.5334/aogh.3578

**Published:** 2022-05-17

**Authors:** Agnes Binagwaho, Brianna Ngarambe, Kedest Mathewos

**Affiliations:** 1 University of Global Health Equity, RW

## Abstract

The term “decolonization” has been increasingly used to refer to the elimination of the colonial experience and its legacy. However, the use of this overarching term masks the real root of the problem. European countries, whose populations are majority white, used their assumed supremacy as justification for the colonization of current low- and middle-income countries (LMICs) where the majority of non-white people live. This clear overlap between geographic and skin color differences explains how the white supremacy ideology triggered European colonization. Therefore, calls to decolonize global health education must focus on the roots of colonization and fight for the elimination of white supremacy ideology that is one of the pillars of the current ills of our global health architecture. A step in this process acknowledging the expertise that emerges from LMICs, alongside challenging the traditional high-income country (HIC) hegemony over knowledge and strengthening universities in LMICs to provide quality medical and global health education. Additionally, we also need to reevaluate curricula, research selection, and design as well as partnerships. Students need to be equipped with the skills to question norms and contribute to the creation of equitable, mutually beneficial partnerships. This needs to accompanied by the adoption of transdisciplinary education to address critical societal challenges. By challenging the white supremacy ideology, we can shift the center of gravity in global health to respect the right to equal say in education and research according to the disease burden and the distribution of the world population.

## Introduction

Within the past few decades, the call for the decolonization of global health has been louder than ever. This was ever more so during the COVID-19 pandemic as the world witnessed the implications of colonization’s lasting legacy. A prime example is the blatant inequity in COVID-19 vaccine distribution and the disregard for the lives of those in low- and middle-income countries (LMICs) as compared to those in high-income countries (HICs) [1]. Many have insisted on the decolonization of global health education as a solution to address such challenges from the onset. This would allow the global health community to rethink certain assumptions and practices early on and ensure that the next generation of global health professionals are well equipped to address these issues when in the field [2].

## “Decolonization” – An overused, inconsequential term

The term *decolonization* was first defined by a German economist, Moritz Julius Bonn, as the process by which countries achieved self-governance [3]. It was used to describe the political phenomenon of independence and was concerned with the “creation of self-governing nation-states” [3]. Later, historians argued that the definition of this term was broadened to cover the elimination of all consequences of the colonial experience, be it political, cultural, economic, or psychological. In fact, it was later used to refer to the removal of supremacy and privilege that are manifestations of the legacy of colonialism and are linked to geography and skin color [4,5].

However, the cause-and-effect relationship needs to be reframed. Colonization and its lasting legacy are products of a mindset that allowed Europeans to justify enslavement, theft of land and wealth from colonies, the destruction of rich cultures and history and the disenfranchisement of colonized populations. Thus, to tackle the problem we need to address its root – the underpinning supremacy mindset initially exercised by Europeans over four centuries ago. Following the forceful geographic expansion of Europeans with white skin color into regions populated largely by individuals with non-white skin color, this European supremacy has become synonymous with white supremacy. While the initial manifestations of this white supremacy mindset have been eliminated with the achievement of political independence in the twentieth century, we see clear evidence of this detrimental mindset through the inequities between LMICs and HICs, both in global health and beyond.

White supremacy today relies on obvert and subtle forms of systemic violence that maintain the privilege that white people consciously or sub-consciously enjoy at the expense of those with non-white skin color [6]. This white supremacy mindset has resulted in the creation of a system that provides white people with better access to education, health, security, housing, loans, fair justice systems etc [7,8]. Given that this mindset is the root of the problem, the term *decolonization* should be replaced by “elimination of the white supremacy mindset”. Focusing on the concept of decolonization without tackling the reality of white supremacy first is a failure to recognize its fundamental role in the exploitation of non-white populations for more than 400 years. Moreover, using the overarching term of decolonization is, for many white people, an excuse to avoid tackling the root cause which is the white supremacy mindset. Thus, attempts to decolonize global health education must focus on eliminating the white supremacy mindset from its delivery through various structural reforms.

## Acknowledging expertise in LMICs

With colonization came the exclusion of individuals from the global South from knowledge-generating bodies and the erasure and abandonment of knowledge and expertise produced in LMICs [9]. This colonial epistemicide contributed to a hegemonic, Eurocentric way of thinking and knowing that continues to undermine LMIC researchers and their contribution to global knowledge [10]. Often, knowledge that emerges from LMICs is not considered sophisticated or rigorous enough to be recognized as evidence. Yet, when HIC researchers study LMIC experiences, ignoring the LMIC experts working in the field, they are recognized as the “global” experts and they consequently benefit from such discoveries [11]. A contributor to this problem is the shortage of research funds in LMIC government and institution budgets, partly driven by the exploitation of resources in LMICs and the intentional decision of the colonial administration to avoid building research infrastructure. This gap is to some extent filled by funding from HIC governments and institutions that often dictate the research agenda and the implementation of health programs. The underlying assumption is that individuals from HICs who are distant from the problems and the communities they are studying are better equipped to lead these research projects than their counterparts in LMICs [12].

There have been some efforts to shift this approach to knowledge production and dissemination. We can take the example of the attempts to integrate indigenous know-how into health service delivery as an example. Dating back to 1978, the World Health Organization’s Health for All Declaration (1978) brought attention to the need to include local people, their traditions and practices in Primary Health Care (PHC) [13]. However, these efforts are insufficient as indigenous knowledge is still deemed ill-advised, uninformed and unscientific by HICs who hold the power that governs global heath. Broadening what is traditionally considered evidence and exposing students both in LMICs and HICs to other ways of learning through books, theories and increased representation of indigenous faculty will contribute to addressing the disregard for knowledge emerging from LMICs or from indigenous communities in HICs. This will challenge the common narrative that knowledge always from HICs to LMICs and ultimately increases the availability of culturally sensitive solutions in the face of challenges to human development.

Addressing power asymmetries in global health education also calls for offering resources, training programs, publications, and conferences in numerous languages as most are only offered in English, French, or Spanish – all of which are colonial languages [14]. Institutions hosting these conferences or trainings should be conscious of the language of their target audience, providing the resources necessary for translation services when needed. This also applies to academia, where the same predominant languages are commonly used in higher education, and as we go up to higher levels of education, linguistic diversity decreases significantly. Peer-reviewed publications that are based in the global North and are considered the epitome of scientific knowledge also largely publish in English, disregarding knowledge generated from scientists who speak other languages and limiting their access to information [15]. Journals such as the *BMJ* should make a conscious effort to include articles written in other languages and provide translation services as needed. While some countries are now pushing for publications in national languages [16], we are far from increasing the reach of such scientific journals. We must highlight the importance of linguistic diversity and diversity in general in universities, as representation from different corners of the world is another approach to shift ways of learning and challenge the white supremacy mindset.

## Location of global health education

A critical challenge within LMICs is the shortage of highly trained professionals who can address the disease burden, contribute to research findings and build, manage and repair equitable healthcare systems. The African continent bears 24% of the global burden of disease and contributes to just 1% of the world’s research output but is home to 3% of the world’s healthcare workforce [17,18]. Various factors contribute to this gap – the refusal to build modern clinical education systems during the colonial period, the resulting shortage of medical and global health academic institutions, brain drain ever since clinical education was established in LMICS, and limited investment in human resources for health being among them [19,20].

Compounding this lack of health sciences training opportunities is the concentration of global health courses in HICs. Eighty-eight percent of global health Master’s programs are located in HICs – countries that built their centers of academic excellence through wealth built from the exploitation of their former colonies. This disproportionate distribution is partly why 83% of leaders in global health come from HICs that represent just 17% of the world’s population [21]. Many of these courses are inaccessible to students in LMICs due to costs associated with tuition, visas, travel, and other living expenses [22]. Instead, these institutions are largely dominated by individuals from HICs who are automatically expected to be superior in skills and in knowledge to their counterparts in LMICs, with research topics determined by them and their funders [23]. Eliminating grant contingencies, having local principal investigators and providing equitable salaries to researchers are some strategies that can be used to address this challenge. Additionally, including experiential learning components within the global health curricula in HIC institutions will expose them to the context they will likely work in and to the challenges that make global health currently inequitable.

As we look towards shifting the power in global health to make it more equitable between HICs to LMICs, it is critical that we invest in the capacity of existing global health institutions in LMICs such as the University of Global Health Equity [24]. Strengthening institutions in LMICs through financial investment and equitable partnerships for research and program development is the best sustainable solution. With investments made to such institutions, we can educate more people than when students from LMIC are supported individually to get educated in HICs at the high risk of them remaining there. Some strategies to strengthen such institutions include faculty exchange programs, programs that provide targeted training based on the needs of the institution and funding directed at improving the institution as a whole rather than one-time fixes. By challenging the white supremacist narrative that knowledge always flows from HICs to LMICs and that quality institutions do not exist in the latter, we can support knowledge generated on the African continent that will enhance the continent’s capacity to respond to diseases and build resilient and equitable health systems. Recent examples include the COVID-19 response outcomes in 2020 and 2021 that proved that Africa, with limited external support, performed better than HICs.

## Embedding research in curricula

Universities play a critical role in knowledge creation. Not only are they tasked with training quality global health clinical and program managers professionals, universities also conduct research that can contribute to solving critical health challenges. More recently, the responsibility of universities in helping achieve the Sustainable Development Goals (SDG) agenda has been emphasized [25]. They can provide the evidence needed to address existing and emerging challenges and enable students to successfully do so. This requires universities to co-create research projects with local communities to identify priority areas instead of adopting a top-down approach which is commonly used in global partnerships. To instill this approach and ensure global health leaders have the humility to learn from the communities they work for, community-based education programs must be embedded within university programs. Moreover, to durably fix weak health systems, universities need to provide evidence to support structural solutions rather than quick one-time fixes. Note that weak health systems in LMICs are an aftermath of colonialism during which services were designed to solely protect the wellbeing of the colonizers and when extended to the local population, were only aimed at keeping the workforce healthy enough to increase production at low cost [26,27]. The resulting weak public health institutions have contributed to poor governance and leadership, inability to deliver public goods as well as preventable suffering and deaths. Research grants provided to universities should incorporate criteria that evaluate the contribution to addressing such structural issues.

Despite the increase in the number of universities in LMICs, many do not incorporate research as a core component of their curricula due to a lack of human and financial ressources [28]. Research can contribute towards the academic growth and overall improvement of students [29], and can enable them to innovate solutions to existing global health problems. While not all research encourages students to challenge the status quo, the essence of research is that it can train students in the critical thinking skills needed to accurately examine a situation and form their opinions. The ability to do so is critical to using local and regional knowledge and challenging the white supremacist mindset. For instance, research can enable students to critically evaluate the fairness of a partnership and examine whether it can contribute to better health outcomes or not. It can also invite students to rethink the meaning of terms such as *Global North, expert, cost efficiency, priority, Global South*, and so on, as these are based on a white supremacist understanding of the world. Looking specifically at the terms *LMIC* and *HIC*, we can see that these classifications are based on Western calculations of what is considered important, that is, monetized materiality of exploited wealth, disregarding cultural wealth, creative wealth and capabilities [30,31,32]. Imbedding this critical thinking at all levels by introducing diversity in theories, books, and lecturers is essential to ensure that the next generation of global health professionals will not continue to be influenced by the white supremacy ideology and will instead promote equal consideration of all stakeholders.

A critical component of global health research that needs to be incorporated into university curricula is implementation research (IR). IR is defined as “the scientific study of the use of strategies to adopt and integrate evidence-based health interventions into clinical and community settings to improve individual outcomes and benefit population health” [33]. It bridges the gap between the discovery of evidence-based interventions, their integration into policy frameworks, their successful implementation and the improvement of health outcomes [34]. A major contributor to inequity in global health are limitations to widespread implementation of known EBIs. Even after discovery, we know that over 50% of EBIs do not reach widespread clinical usage [35]. Current power dynamics between LMICs and HICs exercised through bilateral and multilateral relations, agreements, and obligations also contributes to this failure in equitable implementation. For example, despite the discovery of the COVID-19 vaccine in record time, we have seen vast inequities in COVID-19 vaccine access both within and across countries [36,37]. By equipping LMIC and HIC students with the skills to conduct IR, universities can contribute to tackling vast inequities in healthcare delivery of available health tools – inequities that are often the result of a world based on the white supremacy mindset that attributes lower value to the lives of non-white people. Universities can do this by incorporating equity and IR into curricula, hiring faculty that specialize in IR and availing funding for IR.

## Equitable university partnerships

Partnerships between universities in HICs and LMICs are often not equitably beneficial. While there is no universal model of partnerships, there are universal principles to establishing respectful and mutually beneficial partnerships that give fair credit and ownership of the collaboration to LMIC institutions. A clear manifestation of white supremacy is the persistent habit of institutions in HICs to lead efforts to tackle issues in LMICs, even if the implementers are from LMICs. Moreover, professionals from LMICs are often underpaid in comparison to their counterparts from HICs working on the same project [38]. This is driven by the desire to keep alive the white supremacy agenda and regulate the use of this money, with the goal of leaving out LMIC institutions.

An example is the 30 million USD grant awarded by the US government’s President’s Malaria Initiative (PMI) to PATH, a nonprofit health organization, to work with entities in LMICs who are experienced enough to do it all alone [39]. The PATH consortium has partners from higher education institutions in the US, the UK, and Australia that received the grant to “help” African countries to control and eliminate malaria. Not one African institution, where high-caliber researchers and implementers experts with real knowledge of malaria response work, was included in this US donor’s agenda [39]. Unwillingness by HIC institutions to strengthen LMIC institutions and to prioritize investments where the local disease priority lies, stems from a white supremacy mindset and ultimately leads to a decision-making power imbalance and a lack of sustainable joint coordination.

On the other end of the spectrum, a good example of a partnership that prioritizes the objective of sustainable capacity-building in LMICs is the Human Resources for Health program in Rwanda. This partnership between the Government of Rwanda through the Ministry of Health, the University of Rwanda, and several U.S. institutions funded by the President’s Emergency Plan for AIDS Relief (PEPFAR), was led and managed by the government of Rwanda to increase the quantity and quality of healthcare professionals [40]. The training programs and curricula increased local knowledge and capacity to deliver quality education, using a faculty twinning model and investing in equipment for health services in teaching facilities. The success of the program was evident from the creation of eight residency programs, the education of three times the number of doctors per year, and the fivefold increase in the number of advanced nurses by the end of the program [41]. This partnership is a unique example of the success that can be achieved when we build collaborations that challenge the white supremacy mentality by giving ownership to LMICs.

## Transdisciplinary approach

Historically, certain disciplines have been given more emphasis within universities, resulting in a siloed approach to medical and global health education and practice. For instance, the humanities within public health are often undermined in favor of pure clinical medicine. This disregard of biosocial sciences was common during the colonial era as a disease-specific approach to stop the spread of diseases was considered cheap and good enough for indigenous populations and more feasible to implement rather than improving the overall social and economic factors contributing to illness in colonies [26]. This approach still persists today and prevents healthcare professionals from addressing the needs of a patient comprehensively and from breaking the vicious cycle of poverty and disease for the poor in HICs, majority of whom are non-white, and for LMICs [42]. Note that societal issues such as lack of access to quality healthcare are multisectoral and can only be solved through a strong transdisciplinary, integrative approach. We can take the example of political challenges that compound with health threats and help the spread of infectious pathogens such as SARS-COV-2, resulting in, for instance, the increased vulnerability of refugees during this pandemic.

However, educators and departmental leaders often encounter difficulties obtaining funding for transdisciplinary education and research as most funds are allocated to specific programs or departments. This approach to funding promotes a culture of uni-disciplinarity as faculty and departments are typically ranked and are asked to identify by subject and discipline [43,44]. Furthermore, many funding agencies tend to provide funding to proposals that fall into neat discipline categories [45]. As a result, the majority of transdisciplinary teams have to find unconventional ways to fund their research. To foster transdisciplinary education and research, funding organizations must step away from this siloed approach and even go further to require transdisciplinarity as a component of grant applications. A practical way to do this is to recognize and finance faculty and projects that contribute to driving positive team clinical practice and social change. Given that addressing quality clinical outcomes and societal challenges such as barriers to universal health coverage necessitates a transdisciplinary approach, requiring faculty to explain how their project will address such a challenge will promote transdisciplinarity. This will also result in the respectful inclusion of researchers and implementers from LMICs and require more collaboration across countries and continents – all factors that will contribute to putting an end to the white supremacy mentality [45].

## Conclusion

Various forms of supremacy exist in societies across the globe. However, the white supremacy mindset based on geographic location and is established on differences in skin color, affects all corners of the globe and has wide-reaching impacts in all aspects of society. The removal of white supremacy from global health education is a timely issue that, if successful, can have significant repercussions for the health and wellbeing of the vulnerable across the world. However, because the term *decolonization* is used instead of the phrase “eliminating white supremacy,” which is the primary cause of colonization and of the divide between HICs and LMICs, the global health community fails to tackle the root cause of the problem and consequently delays the solution. This is why undoing the white supremacy mentality and its influence should be our priority as universities working towards eliminating the legacy of colonization in global health education. This includes challenging the white supremacy mentality that governs research and curricula, partnerships and the attitude that ignores the capacity, knowledge, and contributions of LMICs. These actions need to be driven by a genuine commitment to challenging the white supremacy mindset within global health to successfully address the colonial legacy.
